# A rational approach to heavy-atom derivative screening

**DOI:** 10.1107/S0907444909053074

**Published:** 2010-03-24

**Authors:** M. Gordon Joyce, Sergei Radaev, Peter D. Sun

**Affiliations:** a Structural Immunology Section, Laboratory of Immunogenetics, National Institute of Allergy and Infectious Diseases, National Institutes of Health, 12441 Parklawn Drive, Rockville, Maryland 20852, USA

**Keywords:** heavy-atom derivativization, heavy-atom screening, phasing, structure determination

## Abstract

Despite the development in recent times of a range of techniques for phasing macromolecules, the conventional heavy-atom derivatization method still plays a significant role in protein structure determination. However, this method has become less popular in modern high-throughput oriented crystallography, mostly owing to its trial-and-error nature, which often results in lengthy empirical searches requiring large numbers of well diffracting crystals. In addition, the phasing power of heavy-atom derivatives is often compromised by lack of isomorphism or even loss of diffraction. In order to overcome the difficulties associated with the ‘classical’ heavy-atom derivatization procedure, an attempt has been made to develop a rational crystal-free heavy-atom derivative-screening method and a quick-soak derivatization procedure which allows heavy-atom compound identification. The method includes three basic steps: (i) the selection of likely reactive compounds for a given protein and specific crystallization conditions based on pre-defined heavy-atom com­pound reactivity profiles, (ii) screening of the chosen heavy-atom compounds for their ability to form protein adducts using mass spectrometry and (iii) derivatization of crystals with selected heavy-metal compounds using the quick-soak method to maximize diffraction quality and minimize non-isomorphism. Overall, this system streamlines the pro­cess of heavy-atom compound identification and minimizes the problem of non-isomorphism in phasing.

## Abstract 2

In order to overcome the difficulties associated with the ‘classical’ heavy-atom derivatization procedure, an attempt has been made to develop a rational crystal-free heavy-atom-derivative screening method and a quick-soak derivatization procedure which allows heavy-atom compound identification.

## 1. Introduction   

The use of heavy-atom phasing still remains a major technique in *de novo* macromolecular crystal structure determination. However, there are a number of difficulties associated with the technique which have limited its widespread use in recent years. The traditional method usually entails the soaking of multiple crystals in numerous heavy-atom compound solutions for days to weeks (Blundell & Johnson, 1976 ▶). The success of a derivatization is then evaluated through X-ray diffraction data analysis. While the method has been highly utilized in the past, it is too inefficient to support the demands of modern crystallography. The obvious difficulties in the conventional heavy-atom derivative-screening process are that (i) it is an empirical hit-or-miss process based on random screening of numerous heavy-atom compounds, (ii) it requires multiple crystals and (iii) it is a lengthy process requiring multiple X-ray data acquisitions and analyses. The expectations of high-throughput structure determination demand a new, rapid and rational heavy-atom screening procedure. Additionally, the ever-increasing application of crystallo­graphy to difficult projects with often limited amounts of protein samples and crystals make the lengthy routine screening of heavy-atom derivatives impractical.

Here, we summarize the development of a rapid rational procedure for the identification of heavy-atom compounds for phasing. Specifically, we have developed an approach to enable the selection of heavy-atom compounds based on known reactivities in specific crystallization conditions (Agniswamy *et al.*, 2008 ▶). Mass spectrometry is then used to provide a reliable, rapid and crystal-free method for assessing the likely heavy-atom compounds for derivatization (Sun & Hammer, 2000 ▶). A quick-soak method is then used to minimize non-isomorphism and maximize the phasing power of heavy-atom derivatives (Sun *et al.*, 2002 ▶; Sun & Radaev, 2002 ▶).

## 2. Selection of reactive heavy-atom compounds based on their reactivity profiles   

The heavy-metal compounds used in crystallography are generally classified as either class A or class B (Blundell & Johnson, 1976 ▶; Blundell & Jenkins, 1977 ▶). Class A heavy-metal compounds, such as the lanthanides and actinides (primarily uranium), tend to bind to electronegative protein ligands through charge interactions, *e.g.* UO_2_^2+^ binds to the carboxylate group of glutamate and aspartate, as seen in the heavy-atom-bound insulin structure (Blundell *et al.*, 1971 ▶) and also in the prealbumin structure (Blake *et al.*, 1974 ▶). In contrast, class B metals such as platinum, gold and mercury bind covalently to reactive amines and sulfhydryl groups (Islam *et al.*, 1998 ▶; Rould, 1997 ▶). However, other class B metals such as lead and thallium show a different reactivity and tend to interact with hydroxyl groups. Successful heavy-atom derivatization depends not only on the availability of specific amino-acid ligands in a given protein but also to a great extent on the crystallization conditions. Buffer and pH are known to affect the reactivity and solubility of heavy-atom compounds both through chelating heavy atoms and influencing the protonation state of the reactive groups.

To systematically assess the effect of buffer on heavy-atom reactivities, we carried out a series of derivatization experiments using peptides with a single reactive residue (*e.g.* the methionine-containing peptide GEAG**M**ASAGGAG) and class B heavy-metal compounds. These heavy-atom com­pounds generally form covalent adducts with amino-acid ligands and their reactivity depends less on the tertiary conformation of the ligands. Peptides with a single cysteine, methionine or histidine residue were assessed for reactivity with platinum, gold and mercury compounds, while peptides containing a single aspartate, glutamate, asparagine, glutamine or tyrosine residue were used in derivatization experiments with lead-containing compounds. A total of 43 heavy-atom compounds were tested for peptide reactivity in 12 buffer conditions over a wide range of pH. The results are tabulated in Agniswamy *et al.* (2008 ▶) and can be found at http://sis.niaid.nih.gov/cgi-bin/heavyatom_reactivity.cgi. The database can be used to select compounds that are likely to derivatize a given protein of interest under selected buffer conditions.

As expected, heavy-metal compound reactivities depend strongly on buffer and pH conditions. Overall, MES and citrate buffers are the most and least supportive for heavy-atom derivatization experiments, respectively (Table 1 ▶). Therefore, proteins crystallized under MES buffer conditions are likely to be derivatized by a larger range of compounds than those crystallized in any other buffer. Among the basic pH buffers, reactions carried out in HEPES buffer have a greater success rate than those carried out in Tris buffers. However, depending on the peptide ligands available, heavy atoms may react preferentially in either HEPES or Tris buffer. The pH preference of heavy-metal reactivity is also apparent from this study. Gold potassium bromide, potassium tetrabromoaurate, gold potassium thiocyanide and trimethyllead acetate (TMLA) all show high levels of derivatization at slightly acidic to basic pH values, while potassium tetra­cyanoplatinate, gold sodium thiosulfate, mercury(II) chloride, methylmercury(II) bromide, *p*-­chloromercuric benzoic acid, dichloroethylenediamino­platinate and potassium hexachloro­platinate all react strongly under acidic conditions. It is interesting that K_2_IrCl_6_ and K_2_OsCl_6_ are observed to react con­sistently with the Met, Cys and His peptides in the vast majority of conditions examined, but the percentage of total peptide in a reaction which forms a heavy-atom adduct is consistently lower than that seen for other heavy-atom com­pounds.

Another observation which is clear from the data is that a number of compounds are highly reactive over a broad range of buffer and pH. The 22 most reactive compounds are listed in Table 1 ▶ and they include the seven compounds that were previously identified as highly successful in protein-derivatization experiments (Garman & Murray, 2003 ▶; Boggon & Shapiro, 2000 ▶). Other results that stand out include the observation that Met and Cys can be derivatized by at least four heavy-atom compounds in all buffers (Table 2 ▶). Methionine and histidine residues are the most reactive with platinum compounds, while cysteine preferentially reacts with mercury compounds. Thus, for proteins rich in methionine and histidine platinum compounds should be the first choice for screening, while mercury and gold compounds become the obvious candidates for proteins rich in free cysteines. Most importantly, the pH-dependent and buffer-dependent heavy-atom reactivity profiles enable the user to avoid experiments with compounds that are nonreactive in specific buffers, even in an ideal experimental scenario such as the heavy-atom peptide experiment carried out here.

## 3. Assessment of protein heavy-atom derivatization using mass spectrometry   

To replace the traditional time-consuming heavy-atom screening procedure, we utilized mass spectrometry for heavy-atom derivative screening. This method not only enables rapid selection and optimization of the potential derivatives, but also eliminates the use of crystals, allowing streamlining of the heavy-atom derivatization process. Here, we present two test cases to illustrate the general applicability of this method.

### 3.1. Derivatization of FcγRIII   

**Figure 1:**
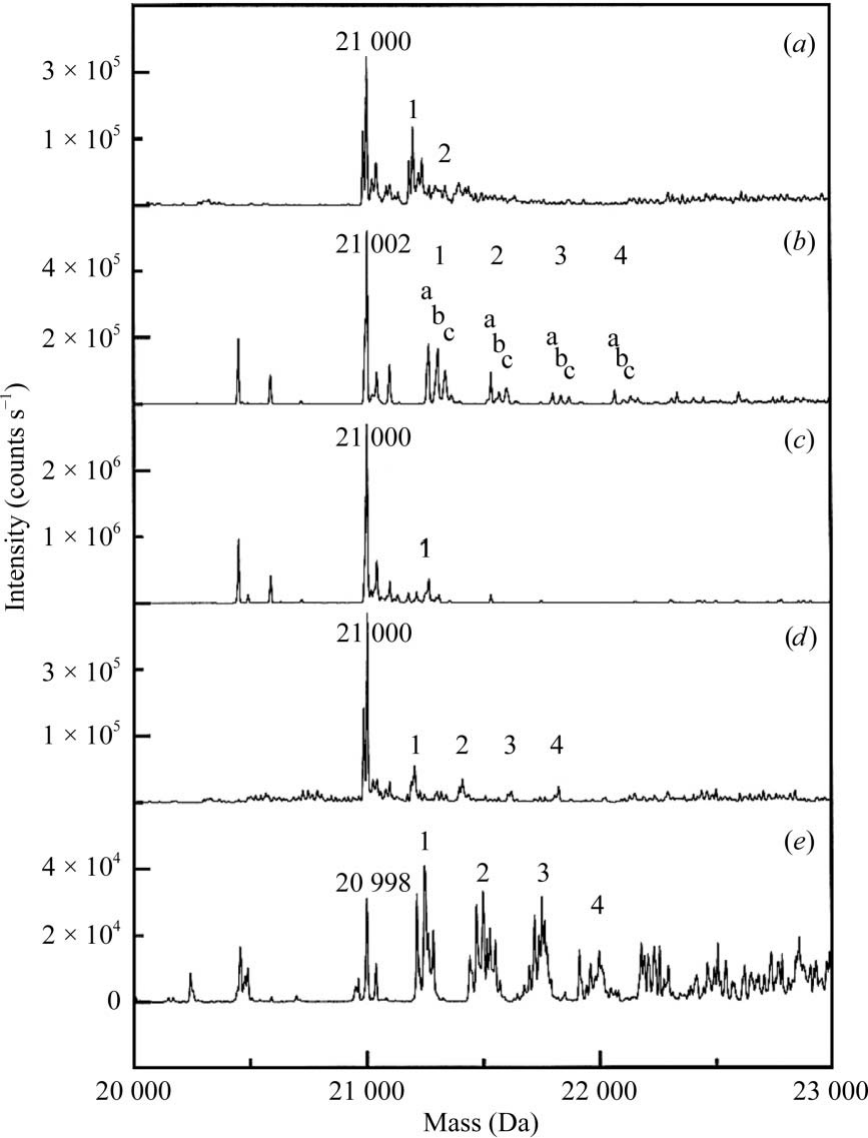
Mass-spectrometric profiles of FcγRIII (calculated molecular weight 20 996 Da) reacted with (*a*) HgCl_2_, (*b*) K_2_PtCl_4_, (*c*) TMLA, (*d*) lead acetate or (*e*) KAu(CN)_2_. The molecular weight of the residual native peak is labeled in each panel. The number of heavy atoms covalently attached to the protein is indicated above the adduct peaks (taken from Sun & Hammer, 2000 ▶).

The extracellular ligand-binding domain of the type III human Fc receptor, FcγRIII, contains two immunoglobulin-like (Ig-like) domains with a molecular weight of 21 000 Da as measured by electrospray ionization mass spectrometry (ESI-­MS). The derivatization reactions were prepared by mixing 0.5–1 µl pre-dissolved heavy-atom compound solutions at various concentrations with 5–10 µl FcγRIII at 2–5 mg ml^−1^ in water for 30 min at room temperature before infusion of the sample into the mass spectrometer. Two adducts of HgCl_2_ with molecular weights of 21 198 and 21 398 Da that corresponded to the addition of one and two Hg^2+^ ions, respectively, were detected in addition to the native peak (Fig. 1 ▶). Additionally, FcγRIII was also found to react with K_2_PtCl_4_, TMLA, lead acetate and KAu(CN)_2_ (Fig. 1 ▶). Furthermore, the numbers of heavy-atom sites found by ESI-MS largely correlated with those found from crystallographic heavy-atom refinement (Sun & Hammer, 2000 ▶).

### 3.2. Derivatization of KIR2DL2   

**Figure 2:**
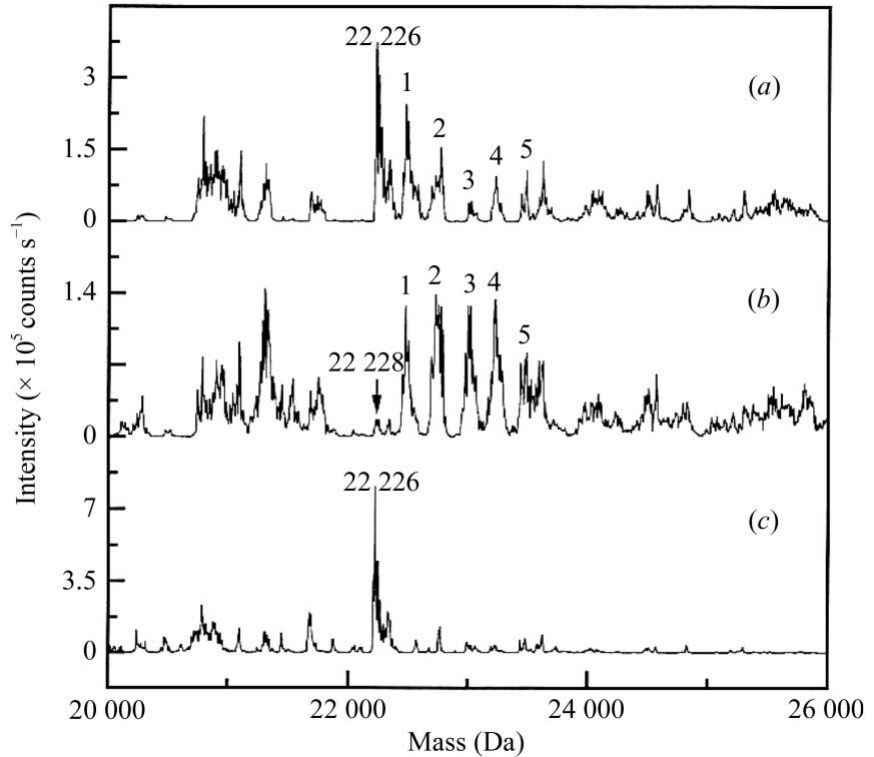
ESI-MS results for gold cyanide-derivatized KIR2DL2. The KAu(CN)_2_-derivatization reaction was carried out using heavy atom:protein molar ratios of (*a*) 9:1 and (*b*) 28:1, respectively. The KAu(CN)_2_-derivatized peaks are labeled 1–5. (*c*) Native KIR2DL2 has a molecular weight of 22 226.0 Da (taken from Sun & Hammer, 2000 ▶).

The extracellular ligand-binding region of KIR2DL2 con­tains two Ig-like domains with a calculated molecular weight of 22 228 Da. The crystal structure of the soluble receptor has previously been determined using KAu(CN)_2_ as the heavy-atom phasing derivative (Snyder *et al.*, 1999 ▶). Two reactions with molar KAu(CN)_2_:KIR2DL2 concentration ratios of 9:1 and 28:1, respectively, were carried out in solution for 30 min using 6.5 µg KIR2DL2 in each reaction. ESI-MS revealed up to five Au(CN)_2_ adducts in addition to the diminished native peak (Fig. 2 ▶). The number of adducts generated by the derivatization reaction in solution agreed with the number of heavy-atom binding sites determined by X-ray diffraction analysis (Sun & Hammer, 2000 ▶). Of the two KAu(CN)_2_ reactions, the reaction with the 28:1 molar ratio of gold cyanide to native protein produced higher derivative-peak intensities than did the 9:1 molar ratio reaction, indicating a correlation between the mass-spectrometric peak intensity and the concentration of the heavy atom used in the derivatization reaction.

In short, mass spectrometry offers a rapid method for heavy-atom derivative screening. Compared with conventional screening by X-ray diffraction, mass spectrometry can be used to screen potential derivatives in solution, thus eliminating the use of crystals. Typical heavy-atom derivatization reactions in solution and mass-spectrometric data acquisition can be completed in minutes to hours, com­pared with the days to weeks required for X-­ray heavy-atom derivative data analysis. The limitation of this mass-spectrometry-based screening technique is that it has only been used for the detection of covalent adducts. It is not clear whether the method can be applied to noncovalently bound heavy atoms such as the lanthanides, although Na^+^, Cl^−^ and other solvent ions are frequently detected as adducts to proteins in mass spectrometry.

## 4. Derivatization by the quick-soak method   

Once heavy-atom compounds with good reactivities in the crystallization buffer have been identified and their ability to react with the protein of interest has been confirmed by mass spectrometry, the process of carrying out heavy-atom soaks with crystals begins. In order to streamline this process and reduce the changes in crystals during the soaking procedure, we have developed a quick-soak method. This method is generally less damaging to the crystals and tends to produce more isomorphous crystals and thus better phasing statistics than conventional soaking techniques. Mass-spectrometric measurements show that adducts of many covalent heavy-atom compounds are formed within minutes in solution (Agniswamy *et al.*, 2008 ▶) and this rapid reaction rate presumably also occurs within crystals. In the following section, we present a comparison of quick-soak-derived phasing statistics with those obtained using conventional longer soaks for crystals of a number of test cases including lysozyme, FcγRIII, the extracellular domain of a type II human transforming growth factor β (TGF-β) receptor (TβRII) and the natural killer cell receptor NKG2D in complex with its ligand ULBP3.

### 4.1. Derivatization of hen egg-white lysozyme crystals   

Two compounds previously known to derivatize lysozyme, KAuCl_4_ and K_2_PtCl_6_, were chosen to identify the optimal time for crystal soaking and the optimal heavy-metal concentrations that should be used. Both of the original derivatives were obtained after 7–14 d of soaking in the heavy-atom solution (Blake *et al.*, 1974 ▶). For the quick-soak method, the lysozyme crystals were soaked in a 10 m*M* solution of heavy-atom compound for 10 min, designated hereafter as the (10 m*M*, 10 min) soak. The data for KAuCl_4_ derivatives were collected from crystals using three different soaking conditions: (10 m*M*, 10 min), (10 m*M*, 24 h) and (1 m*M*, 48 h) (Table 3 ▶). Only the 10 min soak produced diffraction data that were similar in quality to the native data as judged by diffraction resolution, *R*_merge_ and *I*/σ(*I*) for the outermost resolution shell of reflections. Both the 24 and 48 h soaked crystals diffracted to lower resolution than did the native crystal. Interestingly, while the 10 min soaks resulted in the smallest isomorphous *R* factors (*R*_iso_), the heavy-atom occupancies were the highest. Similar results were observed with the 10 min K_2_PtCl_6_ soak, which resulted in no reduction in the diffraction resolution of the lysozyme crystal, whereas once again the 22 and 48 h soaks resulted in weaker diffraction and lower heavy-atom occupancies (Table 3 ▶). When the data from three 10 min soaks with 1, 10 and 12.3 m*M* K_2_PtCl_6_ solutions were compared, the results showed significantly weaker binding of Pt in the 1 m*M* soak compared with the 10 and 12.3 m*M* soaks. This suggests that the quick-soak method optimally requires a higher concentration of heavy-atom solution. While lengthy derivatization reactions ought to result in greater heavy-atom attachment, the observed lower heavy-atom occupancy associated with the longer soaks can be explained by a concomitant increase in non-isomorphism of the crystal arising from the longer soaking time. The lack of isomorphism can also be seen by the change in unit-cell parameters associated with the longer soaks, which is absent in the crystals soaked using the quick-soak procedure.

### 4.2. Derivatives of FcγRIII crystals   

FcγRIII crystallized in space group *P*2_1_2_1_2 and diffracted to 1.8 Å resolution. Both trimethyllead acetate (TMLA) and HgCl_2_ reacted with the receptor as shown by mass spectrometry. Diffraction data were collected from three TMLA-derivatization soaks: (5 m*M*, 10 min), (10 m*M*, 10 min) and (10 m*M*, 24 h). Similar to the lysozyme tests, the (10 m*M*, 10 min) soak resulted in better heavy-atom derivatization than the 24 h soak (Table 4 ▶). A comparison between the two 10 min soaks with 5 and 10 m*M* TMLA showed that the lead occupancies in the (10 m*M*, 10 min) soak are more than twofold higher than those in the (5 m*M*, 10 min) soak, again indicating that the higher concentration of heavy-atom solution has a direct effect on derivatization. For HgCl_2_ soaking, FcγRIII crystals were soaked in saturated HgCl_2_ (less than 5 m*M*) solution for different periods of time. Overnight soaks led to crystal lattice disorder and loss of diffraction. While both the 10 min and 2 h soaks resulted in Hg derivatization (Table 4 ▶), the two major Hg-binding sites in the 2 h soak have higher occupancies than those obtained from the 10 min soak, suggesting that complete HgCl_2_ derivatization took longer than the TMLA-derivatization reaction and that the optimal length of time for soaking may vary depending on the heavy-atom compound and the protein under study.

### 4.3. Phasing of the TβRII structure   

**Figure 3:**
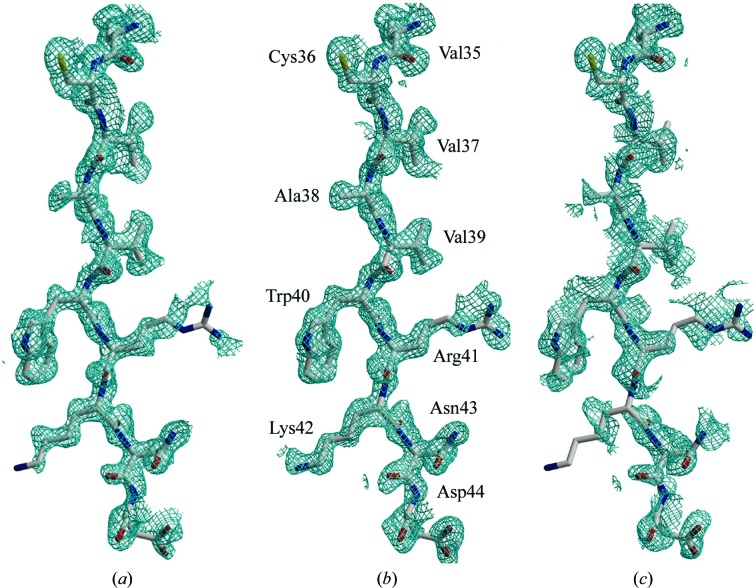
Experimental electron-density maps of TβRII phased with HgCl_2_ derivatives. (*a*) A region of the MAD-phased electron-density map contoured at 1σ with the corresponding refined model. (*b*) SIRAS map produced by a 10 min quick-soak. (*c*) SIRAS map resulting from the long 12 h soak (taken from Sun & Radaev, 2002 ▶).

The extracellular domain of the type II transforming growth factor-β (TGF-β) receptor (TβRII) has been expressed and crystallized (Boesen *et al.*, 2000 ▶). Using mass spectrometry, HgCl_2_ was shown to derivatize TβRII in solution. Crystals of TβRII were derivatized by soaking with saturated HgCl_2_ solution for 10 min and diffraction data were collected around the Hg *L*_III_ absorption edge for structure determination using MAD. For comparison, equivalent MAD data sets were also collected from a crystal derivatized for 12 h using a heavy-atom soaking solution identical to that used in the quick-soak experiment. Overall, the phasing statistics are very similar for both the quick-soak and the 12 h soak, illustrating the effectiveness of the quick-soak in derivatization and subsequent phasing. Again, the calculated *R*_iso_ of the quick-soak derivative (0.23) is lower than that of the longer soak (0.37), indicating increased crystal non-isomorphism as a result of prolonged soaking. This is also reflected in a 1.1 Å change in the unit-cell parameter *a* in the case of the crystal soaked for 12 h compared with a 0.5 Å change in *a* for the crystal soaked for 10 min. Since phases derived from isomorphous replacement (*F*_PH_ − *F*_P_) terms are affected by non-isomorphism between a derivative data set and a native data set, they are often inconsistent with phases derived from anomalous and multi-wavelength components. Attempts to combine these phases often yield electron-density maps that are poorer in quality than those calculated from MAD phasing alone. In this example, the combined phases (SIRAS map) from the shorter soak are not only better than those obtained from the longer soak but they are also better than the MAD phased map, clearly demonstrating the benefits of a quick-soak in reducing crystal non-isomorphism (Fig. 3 ▶).

### 4.4. Phasing of the NKG2D–ULBP3 crystal   

**Figure 4:**
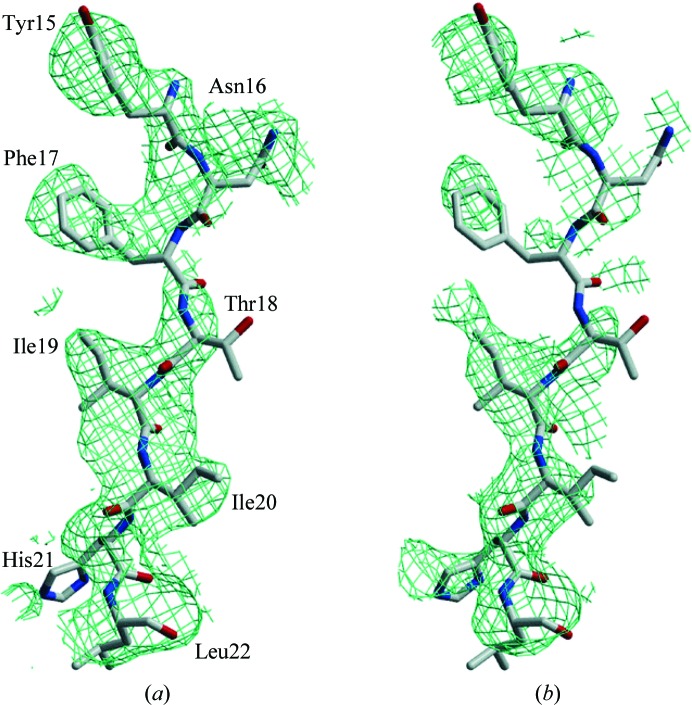
Experimental electron-density maps of the NKG2D–ULPB3 complex phased with a quick-soaked K_2_PtCl_4_ derivative. (*a*) Electron-density map generated from combined MAD and SIR phases contoured at 1σ displaying a β-strand of ULBP3. (*b*) Electron-density map produced from MAD phases alone showing the same region as (*a*) (taken from Sun & Radaev, 2002 ▶).

NKG2D is a 14 kDa C-type lectin-like receptor expressed on the surface of natural killer cells and certain T cells. ULBP3 is a 24 kDa class I major histocompatibility complex antigen-like molecule and a ligand of NKG2D. The crystals of the NKG2D–ULBP3 complex diffracted to 2.6 Å resolution (Radaev *et al.*, 2001 ▶). K_2_PtCl_4_, KAuBr_4_ and KAuCl_4_ showed heavy-atom adducts in mass-spectrometric analysis. Attempts to soak NKG2D–ULBP3 crystals for 24 h in solutions containing 1 m*M* of these heavy-atom compounds all resulted in lattice disorder and loss of diffraction beyond 6 Å resolution. In contrast, a quick-soak of the crystals in 10 m*M* K_2_PtCl_4_ for 10 min resulted in no visual deterioration of the diffraction. A total of four Pt heavy-atom sites were determined and heavy-atom phasing resulted in an overall figure of merit of 0.41. Again, the combined SIR and MAD phases resulted in a better electron-density map than that calculated from the MAD phases alone (Fig. 4 ▶). It is worth emphasizing that only the quick-soak procedure resulted in a usable phasing derivative in this case and that all the longer soaks resulted in large crystal lattice disorder. Thus, the brief soaks are highly advantageous compared with conventional longer soaks for low-resolution diffracting crystals that could easily be damaged by heavy-atom soaks.

Compared with longer conventional soaks, the quick-soak method offers three main advantages. Firstly, it generally preserves the diffraction resolution of a crystal. In all examples tested, the quick-soak derivatization reactions resulted in no obvious deterioration of diffraction resolution compared with that of a native crystal. In contrast, data collected from overnight-soaked crystals often showed a reduction in both resolution and data quality. In some cases, the longer overnight soaks resulted in complete lattice disorder. Secondly, the quick-soak method minimizes the non-isomorphism associated with a derivative data set. This is reflected in smaller unit-cell parameter changes and better phasing statistics in all the quick-soak examples described here. Thirdly, the quick-soak method saves time and offers the potential for high-throughput ‘on-the-fly’ real-time heavy-atom screening.

### 4.5. Choice of heavy-atom concentration and soaking time   

In conventional soaks, the concentration of a heavy-atom reagent is often limited by its adverse effects on the crystal lattice and subsequently the diffraction resolution. These adverse effects are negligible in all four quick-soak test cases described above. Consequently, for the benefit of thorough derivatization, a higher concentration of heavy-atom reagents can and should be used in quick-soak experiments. In both the lysozyme and FcγRIII examples the highest heavy-atom occupancies were obtained with a 10 m*M* or higher concentrations of the heavy-atom reagent. Most of the quick-soak experiments were carried out for time periods between 10 min and 2 h. The optimum soaking time is a balance between achieving high heavy-atom binding occupancy and minimizing crystal non-isomorphism arising from the soaking procedure.

## 5. Applying rational heavy-atom screening to lysozyme   

**Figure 5:**
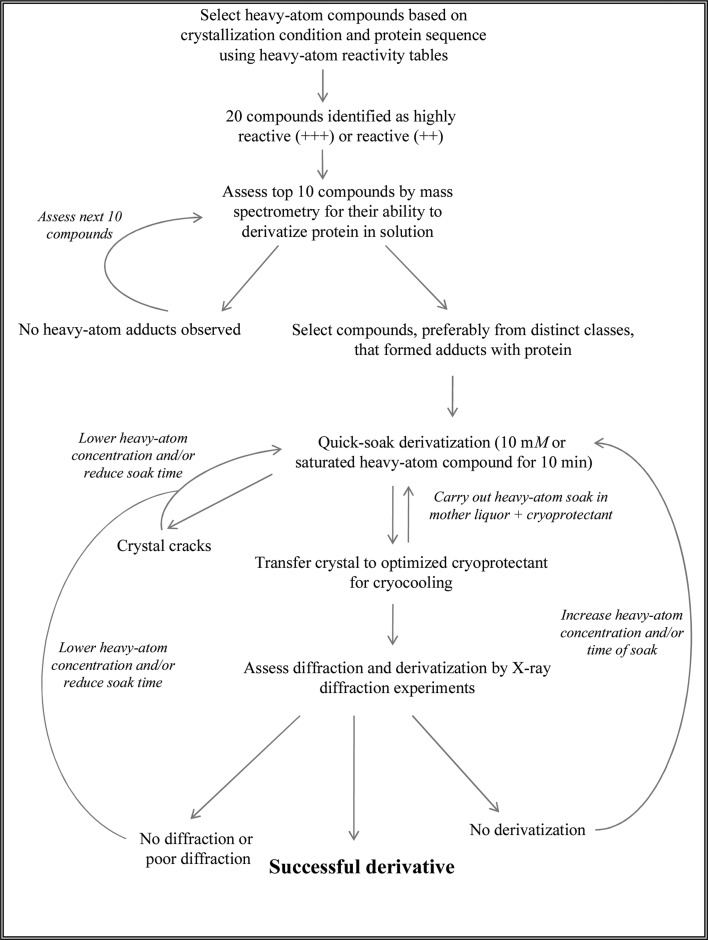
A flow chart outlining the major steps in the rational approach for heavy-atom derivative screening.

The rational heavy-atom screening strategy is summarized in a flow chart (Fig. 5 ▶). As a test case, we applied this rational approach to lysozyme in order to illustrate the gains that can be achieved using this strategy.

Under the crystallization conditions of hen egg-white lysozyme, 15 heavy-atom compounds are predicted to be highly reactive based on the lysozyme amino-acid sequence (Table 5 ▶). Only two of these 15 compounds, K_2_PtCl_4_ and K_2_PtBr_4_, overlap with those phasing derivatives used by Blake (1968 ▶) in the initial structure determination. Several com­pounds known to derivatize lysozyme are not highly reactive with the model peptides in the lysozyme crystallization buffer, suggesting that they may not be optimal for phasing. These 15 heavy-atom compounds were assessed by mass spectrometry to confirm their reactivity with lysozyme. Except for four mercury compounds that were selected based on their reactivities with the cysteine peptide, the remaining 11 compounds all reacted with lysozyme in solution (Table 5 ▶). The failure of the four mercury compounds to derivatize lysozyme is likely to be a consequence of the lack of freely accessible cysteines in the protein. When a protein contains free cysteines they can be highly reactive with many heavy-atom compounds and thus may play a critical role in successful derivatization. In addition, six compounds which failed to react with the peptides in the sodium acetate buffers were selected for test reactions with lysozyme in order to verify that these compounds are less reactive (Table 5 ▶). With the exception of K_2_Pt(CN)_4_, no adduct formation was observed between lysozyme and these test compounds.

**Figure 6:**
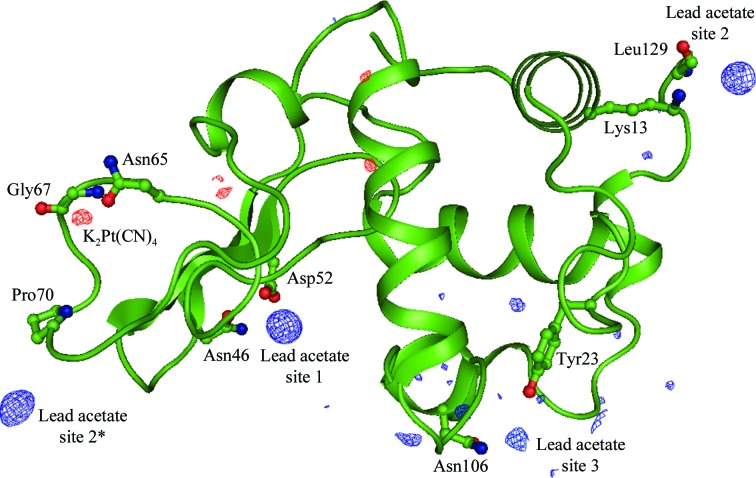
Difference Fourier (*F*_o_ − *F*_c_) maps calculated for lysozyme derivatized with lead acetate (blue density) and with potassium tetracyanoplatinate(II) (red density) and contoured at the 3σ level. The structure of lysozyme is shown in ribbon representation, with the residues coordinating heavy atoms shown in ball-and-stick representation. *PyMOL* was used to generate the figure (taken from Agniswamy *et al.*, 2008 ▶).

Lead acetate, one of the compounds identified as highly reactive in this study but not previously known to derivatize lysozyme, and K_2_Pt(CN)_4_ were used to soak lysozyme crystals using the quick-soak method. The soaked crystals were then analyzed to assess the quality of the data obtained and the extent of derivatization achieved. Three lead-binding sites were identified from the difference Fourier map (Fig. 6 ▶). In contrast, only a minor site was observed in the case of the K_2_Pt(CN)_4_-derivatized crystal. All three lead-binding sites exhibited higher occupancy than the platinum site and the lead derivative also had a higher figure of merit, indicating its potential as a phasing derivative (Table 6 ▶). The results show that while compounds which failed to react with the model peptides may still derivatize a protein in solution, they are likely to produce only minor binding sites in the crystal structure.

## 6. Conclusion   

In summary, it is possible to streamline the conventional heavy-atom derivatization procedure. Use of heavy-atom reactivity profiles allows the rational selection of potential heavy-atom compounds that are amenable to derivatization under experimental crystal-growth conditions. These potential candidates can then be evaluated for their ability to derivatize the target protein by mass spectrometry. In principle, both heavy-atom concentration and soaking time can be optimized using mass spectrometry. Upon verification by mass spectrometry in solution, derivatization reactions in crystals can be carried out using the quick-soak method to minimize non-isomorphism between native and derivatized crystals and thus improve phasing. Overall, the method replaces the most laborious and time-consuming steps in conventional heavy-atom derivatizations with a prediction-based rational approach that should increase the likelihood of successful derivatization and maximize the quality of heavy-atom phases.

## Figures and Tables

**Table 1:** List of the most reactive compounds for heavy-atom derivatization of proteins

Ranking of the most reactive compounds	Derivatization (%)
Ethylmercury(II) phosphate	69.4
Methylmercury(II) acetate	66.6
Sodium tetrachloroaurate	61.1
Potassium tetrabromoplatinate	55.5
Potassium tetrachloroaurate	52.7
Ammonium tetrachloroplatinate	50.0
Gold(III) chloride	47.2
Diaminoplatinum dinitrate	47.2
Thiomersal	47.2
Mercury(II) acetate	47.2
PCMBS	47.2
Potassium tetrachloroplatinate	44.4
Potassium tetranitroplatinate	44.4
Lead acetate	43.3
Potassium hexabromoplatinate	41.7
Methylmercury(II) chloride	38.8
Mersalyl	38.8
Mercury(II) bromide	36.1
Mercury(II) cyanide	33.3
Gold chloride	33.3
Platinum potassium thiocyanate	33.3
Lead nitrate	33.3

**Table 2:** Summary of peptide derivatization The numbers given are for highly reactive compounds which gave greater than 50% derivatization in a single reaction.

	Peptides					
	Met	His	Cys	Asp/Asn/Glu/Gln	Tyr	Total
Sodium acetate	7	4	9	2	3	25
Sodium cacodylate	6	3	11	1	3	24
Sodium citrate	7	0	8	0	0	15
MES	8	26	19	6	4	63
HEPES	7	7	16	5	3	38
Tris	4	3	9	2	2	20
Pt compounds	6	11	3			
Hg compounds	2	7	10			
Au compouds	2	4	3			

**Table 3:** Derivatization conditions and phasing statistics of lysozyme derivatives (adapted from Sun *et al.*, 2002 ▶)

	KAuCl_4_				K_2_PtCl_6_			
Derivatization†	10 m*M*, 10min	10 m*M*, 24h	1 m*M*, 48h	10 m*M*, 10min	12 m*M*, 10min	1 m*M*, 10min	1 m*M*, 22h	10 m*M*, 48h
*R*_iso_	0.201	0.462	0.349	0.176	0.208	0.111	0.087	0.213
Heavy-atom peak height‡								
Site 1 ()	21.6	4.0	15.7	19.3	18.2	5.0	15.0	6.2
Site 2 ()	12.8	4.0	9.3	16.3	16.5	5.0	10.5	11.0
Site 3 ()	9.7	4.0						

† Heavy-atom soaking concentration, soaking time.

‡ The heavy-atom sites are shown as peak heights in standard deviations from the difference Fourier (*F*_PH_ *F*_P_) map. For the KAuCl_4_ derivative the coordinates of sites 1, 2 and 3 are (11.36, 11.72, 19.21), (8.49, 10.2, 14.25) and (3.30, 7.94, 9.84) , respectively. For the K_2_PtCl_6_ derivative the coordinates of site 1 and 2 are (10.957, 10.957, 9.23) and (6.143, 3.859, 29.992) , respectively.

**Table 4:** Derivatization conditions and phasing statistics of FcRIII derivatives (adapted from Sun *et al.*, 2002 ▶)

	TMLA			HgCl_2_	
Derivatization†	5 m*M*, 10min	10 m*M*, 10min	10 m*M*, 24h	Saturated, 10min	Saturated, 2h
*R*_iso_	0.093	0.09	0.168	0.119	0.273
Heavy-atom peak height‡					
Site 1 ()	6.7	17.8	5.0	7.6	24.4
Site 2 ()	6.0	12.8	5.0	5.2	16.4

† Heavy-atom soaking concentration, soaking time.

‡ The heavy-atom sites are shown as peak heights in standard deviations from the difference Fourier (*F*_PH_ *F*_P_) map. The coordinates of sites 1 and 2 of the TMLA derivatives are (111.99, 12.54, 13.41) and (88.49, 21.42, 23.78) , respectively. The coordinates of sites 1 and 2 of the HgCl_2_ derivatives are (80.27, 1.80, 27.71) and (104.52, 7.78, 29.52) , respectively

**Table 5:** Rational heavy-atom screening of lysozyme (adapted from Agniswamy *et al.*, 2008 ▶) The extent of heavy-atom reactivity was evaluated based on the peak heights of observed derivatives from mass-spectrometric experiments and was assigned on a four-level scale as either , +, ++ or +++, which equate to no significant derivative adduct formation and derivative adducts with peak heights less than 25%, between 25 and 50% and above 50% of the native peak intensity, respectively.

Compound	Peptide reactivity	Lysozyme reactivity
MHTS	ND	Blake *et al.* (1962 ▶)
K_2_PdCl_4_	ND	Blake *et al.* (1962 ▶)
K_2_HgBr_4_	ND	Blake (1968 ▶)
K_2_HgI_4_		Blake *et al.* (1962 ▶)
PCMB		Blake (1968 ▶)
PCMBS	+	Blake (1968 ▶)
K_2_PtCl_6_	+	Blake (1968 ▶)
K_2_AuCl_4_	++	Blake (1968 ▶)
K_2_PtBr_4_	+++	+; Blake (1968 ▶)
K_2_PtCl_4_	+++	+++; Blake (1968 ▶)
K_2_PtBr_6_	+++	+++
Methylmercury(II) acetate	+++	+++
Ethylmercury phosphate	+++	+++
Mercury(II) acetate	+++	+++
TELA	+++	+
Lead nitrate	+++	+++
Lead acetate	+++	+++
Diaminoplatinum dinitrate	+++	+
Gold(II) chloride	+++	+
Thiomersal	+++	
Mersalyl	+++	
Mercury(II) bromide	+++	
Methylmercury(II) chloride	+++	
Mercury(II) iodide		
Methylmercury(II) bromide		
K_2_Pt(CN)_4_		+++
K_2_PtI_6_		
Gold sodium thiosulfate		
Hexaphenyllead		

**Table 6:** Phasing statistics of heavy-atom derivatization of lysozyme using two test compounds (adapted from Agniswamy *et al.*, 2008 ▶) Values in parentheses are for the highest resolution shell.

	Lead acetate	K_2_Pt(CN)_4_
Unit-cell parameters ()		
*a*	78.967	77.977
*b*	78.967	77.977
*c*	37.104	36.983
Resolution ()	501.84 (1.911.84)	502.5 (2.592.5)
Completeness (%)	97.4 (94.3)	87.5 (91.6)
*R*_merge_	0.051 (0.165)	0.11 (0.362)
*I*/(*I*)	29.46 (9.77)	11.41 (3.24)
*R*_iso_	0.109	0.319
Figure of merit	0.235	0.144
Heavy-atom peak height (in )		
Site 1	14.6	4.91
Site 2	10.92	N/A
Site 3	5.16	N/A
